# Abnormal keratinocyte differentiation in the nasal planum of Labrador Retrievers with hereditary nasal parakeratosis (HNPK)

**DOI:** 10.1371/journal.pone.0225901

**Published:** 2020-03-02

**Authors:** Jeanette Bannoehr, Pierre Balmer, Michael H. Stoffel, Vidhya Jagannathan, Véronique Gaschen, Kathrin Kühni, Beyza Sayar, Michaela Drögemüller, Denise Howald, Dominique J. Wiener, Tosso Leeb, Monika M. Welle, Eliane J. Müller, Petra J. Roosje

**Affiliations:** 1 Division of Clinical Dermatology, Department of Clinical Veterinary Science, Vetsuisse Faculty, University of Bern, Bern, Switzerland; 2 DermFocus, Vetsuisse Faculty, University of Bern, Bern, Switzerland; 3 Division of Veterinary Anatomy, Vetsuisse Faculty, University of Bern, Bern, Switzerland; 4 Institute of Genetics, Vetsuisse Faculty, University of Bern, Bern, Switzerland; 5 Department of Clinical Research, Molecular Dermatology and Stem Cell Research, University of Bern, Bern, Switzerland; 6 Institute of Animal Pathology, Vetsuisse Faculty, University of Bern, Bern, Switzerland; 7 Clinic for Dermatology, Inselspital, Bern University Hospital, Bern, Switzerland; University of Pisa, ITALY

## Abstract

Hereditary nasal parakeratosis (HNPK) is an inherited disorder described in Labrador Retrievers and Greyhounds. It has been associated with breed-specific variants in the *SUV39H2* gene encoding a histone 3 methyltransferase involved in epigenetic silencing. Formalin-fixed biopsies of the nasal planum of Labrador Retrievers were screened by immunofluorescence microscopy for the presence and distribution of epidermal proliferation and differentiation markers. Gene expression of these markers was further analysed using RNA sequencing (RNA-seq) and ultrastructural epidermal differences were investigated by electron microscopy. Differentiation of the nasal planum in the basal and suprabasal epidermal layers of HNPK-affected dogs (n = 6) was similar compared to control dogs (n = 6). In the upper epidermal layers, clear modifications were noticed. Loricrin protein was absent in HNPK-affected nasal planum sections in contrast to sections of the same location of control dogs. However, loricrin was present in the epidermis of paw pads and abdominal skin from HNPK dogs and healthy control dogs. The patterns of keratins K1, K10 and K14, were not markedly altered in the nasal planum of HNPK-affected dogs while the expression of the terminal differentiation marker involucrin appeared less regular. Based on RNA-seq, *LOR* and *IVL* expression levels were significantly decreased, while *KRT1*, *KRT10* and *KRT14* levels were up-regulated (log2fold-changes of 2.67, 3.19 and 1.71, respectively) in HNPK-affected nasal planum (n = 3) compared to control dogs (n = 3). Electron microscopical analysis revealed structural alterations in keratinocytes and stratum corneum, and disrupted keratinocyte adhesions and distended intercellular spaces in lesional samples (n = 3) compared to a sample of a healthy control dog (n = 1). Our findings demonstrate aberrant keratinocyte terminal differentiation of the nasal planum of HNPK-affected Labrador Retrievers and provide insights into biological consequences of this inactive *SUV39H2* gene variant.

## Introduction

Hereditary nasal parakeratosis (HNPK) is an inherited disorder in Labrador Retrievers (LR) which has been recognized for more than 15 years [1, 2]. Recently, the same clinical and histological presentation of HNPK was described in Greyhounds [3]. Based on pedigree analysis of affected dogs, an autosomal recessive mode of inheritance was determined in LR dogs [1, 2]. Typically, the clinical sign is a non-pruritic hyperkeratosis of the nasal planum in otherwise healthy dogs. Only one publication reported involvement of the bridge of the nose, pinnae and paw pads [1]. Although initial discrete alterations of the nasal planum can be visible in 6–12 weeks old LR puppies, clinical signs become typically apparent at 6–24 months of age and range from mild (dorsal nasal planum hyperkeratosis) to more severe lesions (fissures and erosions) [1, 2]. Treatment options are limited and aim at topical moisturization by daily application of ointments or propylene glycol [4]. More severe cases may require immunomodulatory treatment such as topical corticosteroids or tacrolimus and secondary infections may be an additional complicating factor [4].

The histopathology of HNPK has been well described and features a striking parakeratotic hyperkeratosis interspersed with serum lakes in the corneal layer and stratum granulosum, and cytoplasmic vacuolation (hydropic degeneration) of keratinocytes throughout the epidermis, accompanied by variable degrees of dermal and epidermal (predominantly lymphocytic) inflammation [1, 2, 5].

The exact pathomechanism underlying HNPK in LR and Greyhounds has not yet been identified. A N324K missense variant in the *SUV39H2* gene has been proposed as the genetic cause for HNPK in LR [5]. It was earlier demonstrated that the reported N324K variant in the evolutionary conserved SET domain of *SUV39H2* leads to an inactive SUV39H2 enzyme [6], thus implying a functional role of this variant in HNPK. Interestingly, HNPK in Greyhounds was associated with a splice site variant in the *SUV39H2* gene [3]. *SUV39H2* encodes a histone 3 lysine 9 trimethyl (H3K9me3) transferase, which belongs to the large family of lysine methyltransferases. H3K9me3 epigenetic changes catalyzed by SUV39H2 and other H3K9me3 transferases constitute a chromatin-silencing histone modification, resulting in transcriptional alterations [7, 8].

Epigenetic regulation of transcription ensures correct development in any given cell type and is consequently involved in a number of cellular processes like proliferation and differentiation and chromatin-associated proteins like histone methyltransferases usually regulate a large number of genes and loci throughout the genome [9]. However, the role of SUV39H2 in skin is currently unknown.

In regular epidermal differentiation, keratinocytes passage through the different epidermal layers and develop from proliferating to differentiating and ultimately dying cells [10, 11]. This process is highly ordered and finely tuned with specific protein expression patterns at each stage. Therefore, specific proteins are used as markers for proliferation and epidermal differentiation [11, 12]. They are specific for each level, for example, Ki-67 in the stratum basale, desmoglein 1 in the suprabasal to the outermost viable layers and loricrin, involucrin and filaggrin in terminal stages of differentiation when the cornified envelope (CE) is formed [10, 13]. Further, specific keratins are expressed at different stages of epidermal differentiation, e.g. K5 and K14 in the basal layer, and K1 and K10 in suprabasal keratinocytes [13]. In terminally differentiated cells, loricrin, involucrin and other proteins are cross-linked by transglutaminases and serve as structural proteins for reinforcement of the CE [10]. These proteins are assembled at the inner surface of the plasma membrane of terminally differentiated, anucleate keratinocytes (corneocytes), forming the stratum corneum (SC). In squamous epithelium, the SC results from a balance between formation and desquamation of corneocytes and has variable thickness depending on body site.

Our knowledge about the protein expression patterns in canine epidermis and especially the nasal planum is limited. The epidermis of the canine nasal planum is on average more than twice the thickness of haired skin in healthy dogs and displays an orthokeratotic SC but focal parakeratosis can occur [14]. One study showed that the healthy canine nasal planum displays a similar pattern of the differentiation markers K14 and K10, desmogleins 1 and 3, and filaggrin, compared to a canine *in vitro* three-dimensional skin equivalent [15]. Another study reported on comparable mRNA and protein expression of K5 and K10, filaggrin and involucrin in various haired body regions of dogs [16]. In the nasal planum of LR with HNPK, the pattern of desmoglein 1 and the number of cells positive for the proliferation marker Ki67 is similar to non-affected control dogs [5].

The aim of the current study was to define the differentiation pattern in the nasal planum of LR carrying the *SUV39H2* genetic variant using immunofluorescence (IF) microscopy for selected markers of epidermal differentiation, and to complement IF experiments by RNA sequencing (RNA-seq) and transmission electron microscopy (TEM).

## Materials and methods

### Ethics statement

Biopsies and blood samples were taken with informed owner consent. All animal experiments were performed according to local regulations, and the cantonal committee on animal experimentation Switzerland (permits BE22/07 and BE23/10) approved the work conducted.

### Samples

The database of the Institute of Animal Pathology, Vetsuisse Faculty, University of Bern was searched for LR patients where the diagnosis of HNPK was made based on typical histopathological features. The HNPK-affected group consisted of three male (one neutered, two intact) and three female (two neutered, one intact) LR, with age ranging from 2 to 11 years (median 4.5 years). Biopsies were taken at owners’ request to establish a definite diagnosis of HNPK based on the histopathological picture. The control group consisted of six LR (three males [two neutered, one intact] and three females [two neutered and one unknown]) with a clinically normal nasal planum, confirmed by histopathology. The age of the control dogs ranged from 8 to 14 years with a median of 11.5 years. All dogs in the control group had to be euthanized for unrelated disease and biopsies were taken immediately post mortem. Additional biopsies included for control purposes were from non-lesional abdominal skin and paw pads from two HNPK LR and two laboratory beagle control dogs (euthanized for unrelated reasons).

### Confirmation of the *SUV39H2* genotype in affected and non-affected dogs

The homozygous genotype for *SUV39H2*:*c*.*972T>G* (G/G variant) was confirmed for all HNPK dogs and the homozygous wildtype T/T genotype for the control group on genomic DNA from either deparaffinized tissue or EDTA-blood. The isolation of genomic DNA from paraffin embedded tissue samples was carried out by standard methods using the DNeasy Blood & Tissue Kit (QIAGEN, Hilden, Germany) combining the protocol for pretreatment of paraffin-embedded tissue and the spin-column protocol for purification of total DNA from animal tissue as recommended by the manufacturer. The associated variant was genotyped by re-sequencing of PCR products using Sanger sequencing technology. PCR products were amplified using AmpliTaq Gold 360 Mastermix (Life Technologies, Zug, Switzerland), and the primers SUV39H2_Ex4_FS 5’TCCTCAACTATGGACAAATCGTT3’ and SUV39H2_Ex4_RS 5’TCTCTTTGATCTGGATTATGAATCTG3’. The products were directly sequenced on an ABI 3730 capillary sequencer (Life Technologies, Zug, Switzerland) after treatment with exonuclease I (New England Biolabs, Ipswich, MA, USA) and rAPid alkaline phosphatase (Roche, Basel, Switzerland). Sequence data were analyzed with Sequencher 5.1 (GeneCodes Corporation, Ann Arbor, MI, USA).

### Immunofluorescence staining of deparaffinized tissue slides

Nasal planum biopsies of six HNPK-affected and six non-affected LR were analyzed employing all five differentiation markers. For loricrin, a second set of slides including the nasal planum, paw pads and abdominal skin of two affected dogs and two non-affected control dogs was stained. Formalin fixation and paraffin-embedding of tissue, preparation of slides and hematoxylin and eosin (H&E) staining was performed according to standard methods. IF staining was done as described previously [5]. Slides were mounted with fluorescence mounting medium (DAKO, Glostrup, Denmark) and stored protected from light at 4°C until further analysis. In all experiments, non-specific rabbit or mouse IgG (Santa Cruz Biotechnology, Dallas, TX, USA) was used instead of primary antibody as negative control, abdominal skin biopsies of a non-affected LR as positive control. Data were analyzed semi-quantitatively (microscopic evaluation using Openlab software, PerkinElmer, Waltham, MA, USA). The complete set of slides was processed on the same day to ensure identical laboratory conditions. Each antibody was used individually to exclude cross-reactivity and each experiment was performed twice on different days to ensure repeatability of results.

### RNA isolation and RNA sequencing

Nasal planum biopsies of three HNPK-affected dogs and three control dogs were included for RNA-seq. The nasal epidermis (including the rete ridges) was manually separated from the dermis using a stereo microscope. The epidermis of the nasal planum is very thick and fully pigmented and can be visually discerned from the underlying dermis. These properties together with the difference in texture between epidermis and dermis facilitate separation of the layers by microdissection. Proteases to facilitate dermal-epidermal separation were not used to prevent alterations of the transcription patterns. Total RNA was isolated with the RNeasy fibrous tissue kit (Qiagen, Hilden, Germany). The integrity of the isolated RNA was confirmed on a 2100 Bioanalyzer (Agilent Technologies AG, Basel, Switzerland) and the yield was determined by fluorimetry. Approximately 1μg of total RNA was processed into stranded mRNA libraries using a commercial kit according to the manufacturer’s instructions (TruSeq stranded total RNA sample prep kit; Illumina, San Diego, CA, USA). Six fragment libraries (three control LR and three HNPK- affected LR) with 350 bp insert size were prepared and two lanes of Illumina HiSeq2000 paired-end reads (2 x 100 bp) were collected, obtaining 63,280,070 tags on average per library.

### Data analysis of RNA libraries

Prior to mapping reads to the dog reference genome, we filtered all sequences to remove adaptor sequences and low-quality sequences (sequences with 50 and more bases with quality value less than 15). We mapped the quality filtered reads to the dog reference genome (CanFam3.1) using the splice alignment program TopHat2 (version 2.0.4) with default parameters [17]. We also used a currently curated catalog of improved keratin gene annotations [18] due to high sequence homology or posttranslational alterations in this gene family.

The gene models from Ensembl build 70 were used for read counting using HTSeq-count (version 0.5.3p9). RSeQC (v2.3.3) was used for read distribution over gene body to check 5′/3′ bias [19]. The read counts were used in the EdgeR package for analyses of differentially expressed genes [20]. All data were analysed using the R statistical environment. The significance of differentially expressed genes was identified with a 1% false discovery rate (FDR) and fold change greater than 1.5.

We obtained approximately 55 to 70 million read-pairs for each of the six cDNA libraries. Quality filtered reads were mapped to the dog reference genome canFam3.1 using default parameters of Top-hat software. On average, 80% of the reads were mapped to the reference genome of which 90% were uniquely mapped and 76.5% concordantly aligned (see S1 Table for details). Differentially expressed genes between cases and controls were identified with tools HTseq and DESeq from Bioconductor (http://www.bioconductor.org/). Log2 fold changes comparing the three cases with the three control samples were obtained using EdgeR.

### Transmission electron microscopy (TEM)

TEM was performed on biopsy samples of nasal planum from three HNPK-affected and one healthy dog. Immediately upon collecting the samples, tissue was immersion-fixed with 2.5% glutaraldehyde in 0.1 M cacodylate buffer, pH 7.4 at 4°C overnight. After three washing steps in cacodylate buffer, samples were post-fixed with 1% osmium tetroxide (OsO4) (Chemie Brunschwig, Basel, Switzerland) in 0.1 M cacodylate buffer for 4 h at 4°C and again washed three times with cacodylate buffer. Tissue was dehydrated in an ascending ethanol series and embedded in Epon, a mixture of Epoxy embedding medium, dodecenylsuccinic anhydride (DDSA) and methyl nadic anhydride (MNA) (Sigma Aldrich, Steinheim, Germany). Epon was polymerized for 5 days at 60°C. Resin blocks were trimmed and regions of interest were identified based on semithin sections that had been stained with toluidine blue. Ultrathin sections of 60–70 nm in thickness were obtained with diamond knives (Diatome, Biel, Switzerland) on a Reichert-Jung Ultracut E (Leica, Heerbrugg, Switzerland). Sections were double-stained with 0.5% uranyl acetate for 30 min at 40°C (Sigma Aldrich, Steinheim, Germany) and 3% lead citrate for 10 min at 20°C (Laurylab, Saint Fons, France) in an Ultrastain^®^ (Leica, Vienna, Austria). A Philips CM12 transmission electron microscope (FEI, Eindhoven, The Netherlands) was used for sample examination and micrographs were captured with a Mega View III camera using the iTEM software (version 5.2; Olympus Soft Imaging Solutions GmbH, Münster, Germany).

## Results

### HNPK dogs exhibit altered expression of late differentiation markers

**Fig 1:**
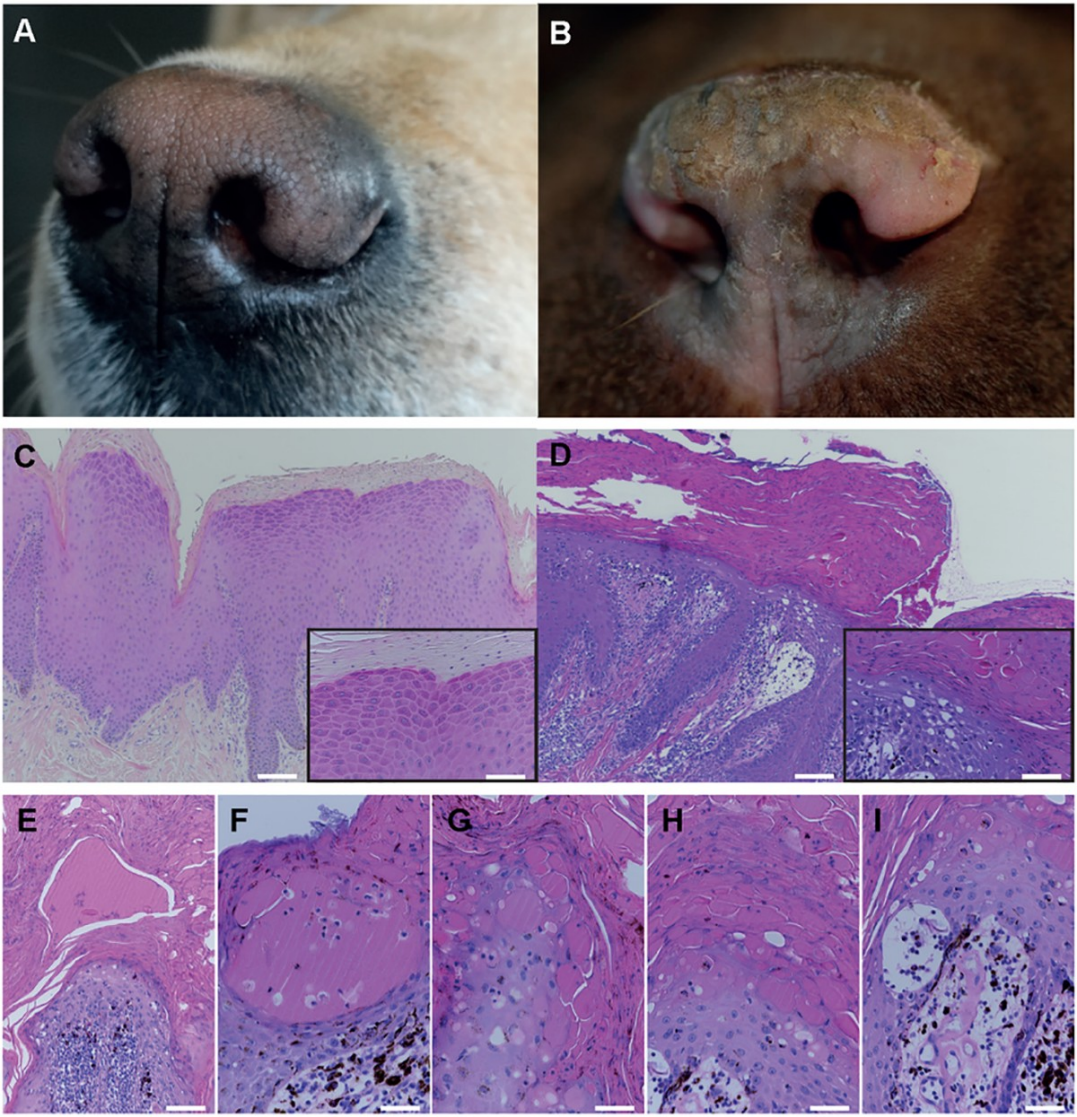
Presentation of the clinical phenotype and histopathological characteristics of HNPK. (A) Nasal planum of a non-affected LR. (B) Nasal planum of an HNPK-affected LR. Note the depigmentation, hyperkeratosis and discrete fissure in the affected area (B) compared to the shiny cobblestone structured surface of the non-affected nasal planum (A). (C-D) Representative H&E-stained paraffin sections of non-affected (C) and HNPK-affected LR nasal planum (D) with insets showing in more detail regular differentiated upper layers of the epidermis and stratum corneum (C), in contrast to the irregular epidermis and stratum corneum of the affected dog (D). (E-I) Representative examples of different-sized serum lakes in the stratum corneum (E), in the stratum granulosum or stratum corneum (F-H), and intra-epidermal vacuoles (H-I) in nasal planum of HNPK-affected dogs. Scale bars are 100μm (C-D), 50μm (E), and 25μm for high magnifications in insets and F-I.

Biopsies from the nasal planum of six clinically affected HNPK LR and six non-affected LR were used to assess the expression pattern of differentiation markers. All HNPK LR were confirmed to be homozygous for the *SUV39H2*:*c*.*972T>G* variant that was absent in the non-affected LR. Routine H&E staining of biopsies showed typical histopathological alterations in HNPK dogs [5]. The epidermis of affected LR was irregular with long rete pegs and parakeratotic hyperkeratosis. Furthermore, prominent serum lakes were characteristically present in the stratum granulosum and SC of these dogs (Fig 1E–1I).

**Fig 2:**
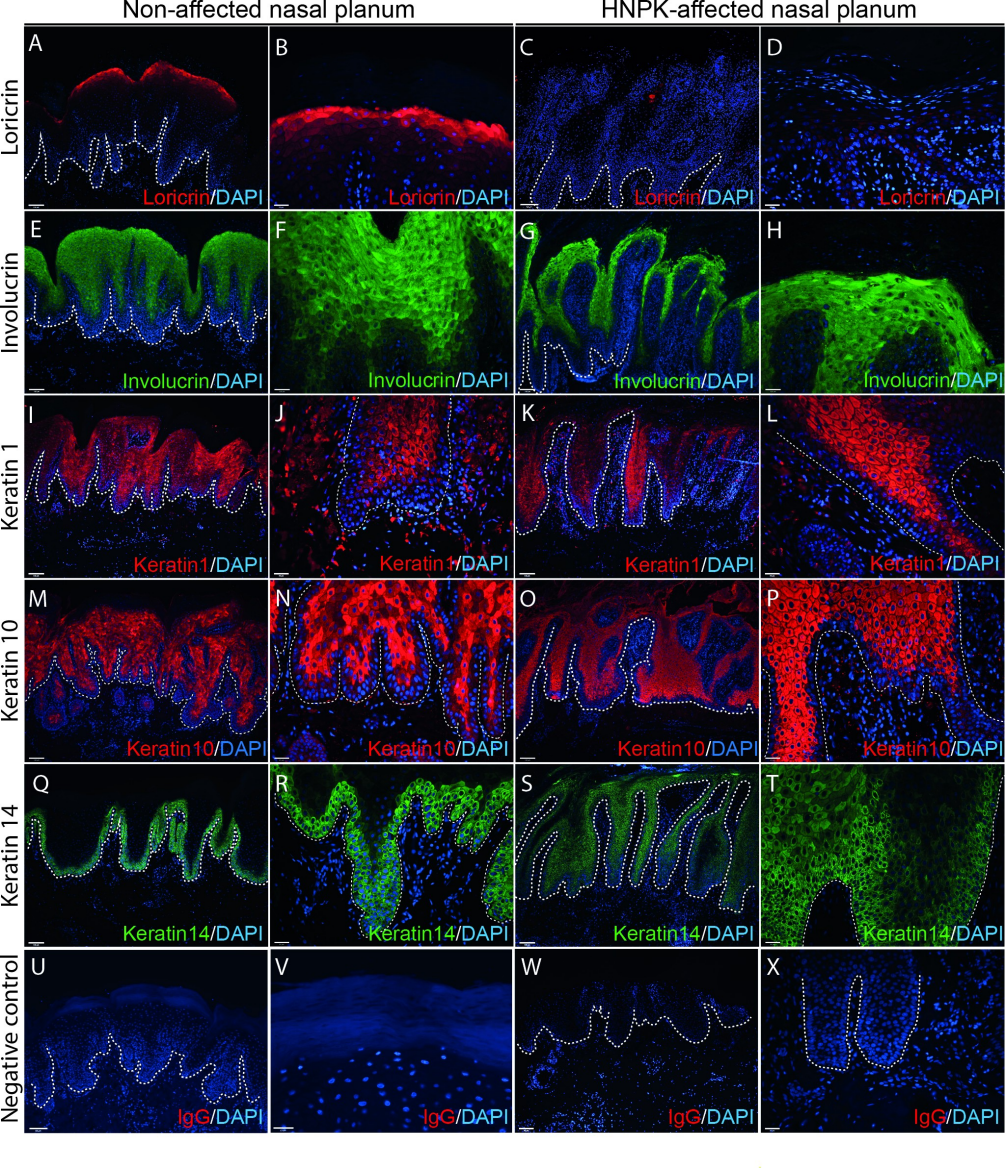
Immunofluorescence microscopy of control and HNPK nasal planum. The individual antibodies used are indicated on the left side of the figure. Normal and HNPK- affected nasal planum staining: loricrin (A-B/C-D), involucrin (E-F/G-H), K1 (I-J/K-L), K10 (M-N/O-P), K14 (Q-R/S-T) and the negative IgG control (U-V/W-X). Each staining was performed in parallel on sections of six non-affected and six HNPK-affected nasal plana and results were reproducible (N≥2). White hatched lines indicate the dermal-epidermal junction. Scale bars are 100μm for low magnification and 25μm for high magnification.

Five differentiation markers were chosen for IF microscopy, mirroring different stages of the epidermal differentiation process: K14 representing basal-, K1 and K10 suprabasal layers, involucrin and loricrin indicating late spinous and/or subcorneal layers, respectively, or terminal stages of differentiation. Loricrin expression could not be detected in any sample of HNPK-affected nasal planum (Fig 2C and 2D) and involucrin expression was delayed, and disorderly compared to the non-affected control dogs (Fig 2E–2H). The pattern of K14, K1 and K10 was not markedly altered in HNPK-affected nasal planum compared to healthy dogs (Fig 2I–2T). Although K14 appeared to be less visible in the basal layers at the tips of the rete pegs of HNPK dogs at low magnification, at higher magnification it was present in a similar pattern starting in the basal layer and comparable to control dogs. K1 and K10 staining began in all dogs above the basal layer. Scale bars are 100μm for low magnification and 25μm for high magnification.

### Altered expression of differentiation markers is restricted to the nasal planum in HNPK dogs

**Fig 3:**
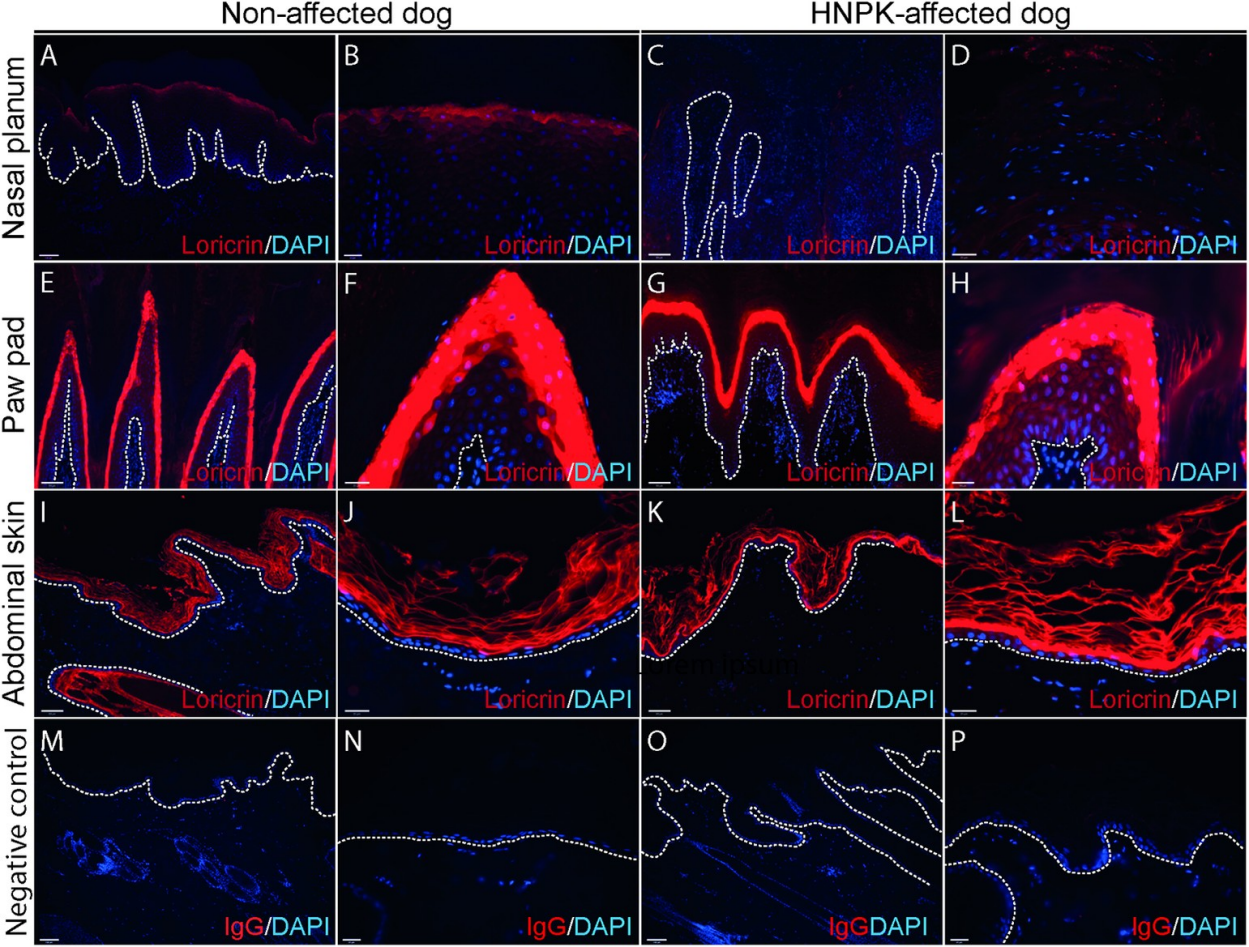
Loricrin staining of the nasal planum, paw pads and abdominal skin of non- and HNPK-affected dogs. Whereas loricrin is present in non-affected nasal planum (A-B), it is absent in nasal planum of HNPK-affected dogs (C-D). However, there is no difference in the loricrin pattern in paw pads (G-H) and abdominal skin (K-L) of HNPK-affected dogs and non-affected dogs (E-F resp. I-J). IgG staining was performed as negative control (M-P). Stainings were performed in parallel on sections of two non-affected and two HNPK-affected dogs and results were reproducible (N≥2). White hatched lines indicate the dermal-epidermal junction. Scale bars are 100μm for low magnification and 25μm for high magnification.

To establish whether loricrin expression was lacking in other anatomical regions of HNPK dogs, paw pad and abdominal skin biopsies of affected and control dogs were examined. Loricrin was present in paw pads and abdominal skin of HNPK-dogs in a pattern comparable with non-affected samples (Fig 3E–3L). Involucrin staining on haired skin did not reveal differences between HNPK-affected and non-affected dogs.

### Expression profiling confirms aberrant differentiation in HNPK

**Fig 4:**
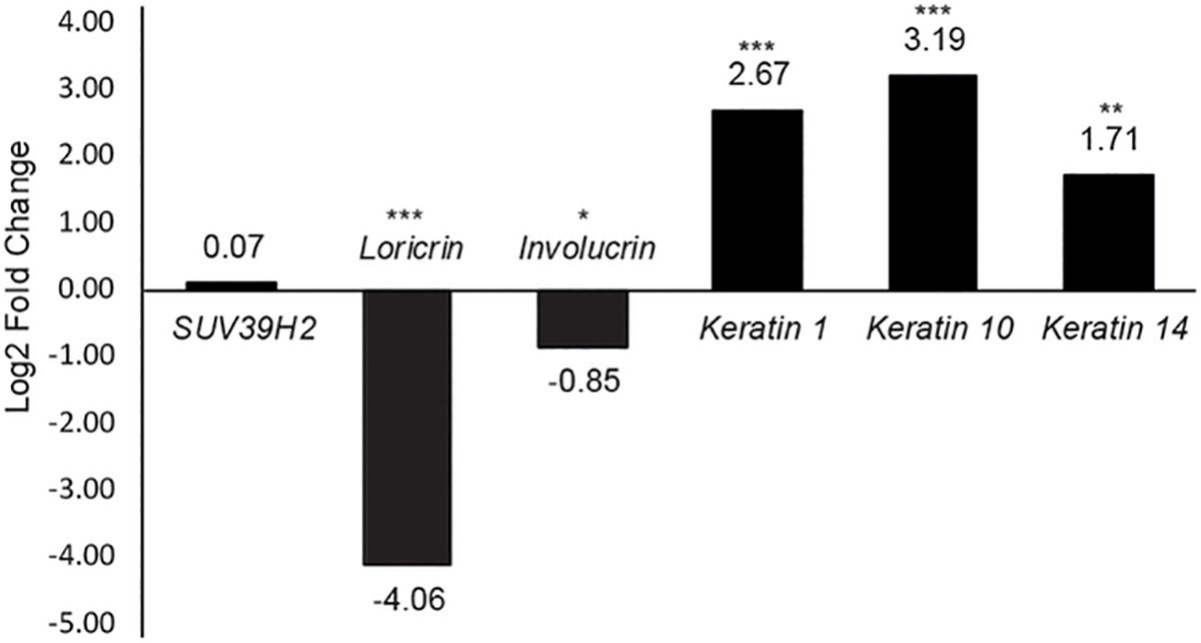
Differential gene expression comparing nasal planum of HNPK-affected and non-affected dogs. Notice that *SUV39H2* log2fold change is not significantly different between the two groups (HNPK-affected dogs (N = 3) resp. control dogs (N = 3)). Selected differentiation markers (*LOR* and *IVL*) and *KRT1*, *KRT10* and *KRT14* are depicted according to RNA-seq data. Numbers above or below bars indicate the log2fold change. *P <0,05, **P < 0.001, ***P <0.0001.

Using EdgeR, a total of 1929 genes were shown to be significantly (p<0.05) differentially expressed between cases and controls at log2 fold change >1 (40.54% of up-regulated genes) and a log2 fold change <-1 (59.46% of down-regulated genes). The *SUV39H2* gene expression was not significantly different between HNPK-affected and control dogs. Furthermore, *LOR* expression had a log2fold-change of -4.06 in HNPK-affected nasal planum, indicating a highly decreased gene expression. *IVL* expression was also significantly down-regulated in HNPK-affected nasal planum, with a log2 fold-change of -0.85, but *KRT1*, *10* and *14* showed log2fold-changes of 2.67, 3.19 and 1.71, respectively, presenting an up-regulated gene expression of investigated keratins in HNPK-affected nasal planum. In Fig 4, these log2 fold changes are depicted, comparing three cases with three control samples.

### Ultrastructural changes occur in superficial epidermis in HNPK

Lymphocytes were few in both control and HNPK samples and were predominantly located below or in the basal layer of the epidermis. Tonofilament bundles in intermediate epidermal layers (spinous and granular layer), desmosomes and keratohyalin granules were equally abundant and typical in both healthy and HNPK samples. Lamellar bodies could be identified but were few in a control sample and only rarely seen in HNPK samples.

**Fig 5:**
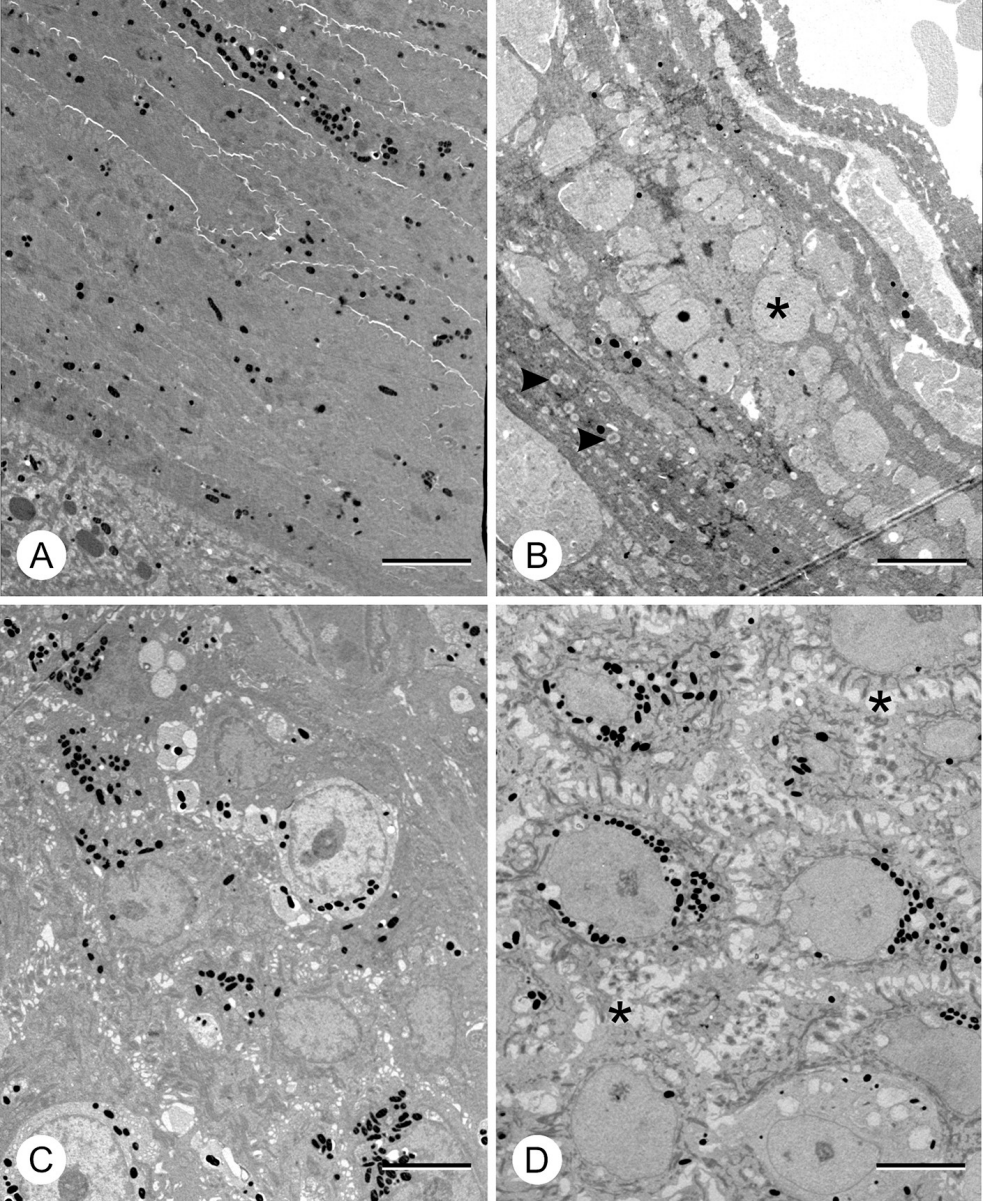
Transmission electron microscopy of nasal planum sections. Transmission electron micrographs showing stratum corneum (A-B) and stratum spinosum (C-D) of healthy control animals (A and C) and HNPK-affected dogs (B and D). In superficial layers, intercellular space is distended in HNPK-affected dogs (B and D: asterisks) as compared to healthy individuals. Dense core granules (B: arrowheads) were only noted in affected animals. Scale bars = 5 μm.

In the superficial cell layers of the HNPK samples, the intercellular space (Fig 5D: asterisks) was conspicuously widened between desmosomes, and neighbouring cells remained connected through thread-like cellular bridges only (Fig 5D). Eventually, these cell contacts were lost and cell layers were separated by large clefts. The prominent serum lakes as seen in the stratum granulosum and corneum by optical microscopy turned out to be these severely distended regions of the intercellular space. They were filled with a homogeneous, finely granular electron-lucent material and sometimes contained erythrocytes. Prominent granules with a dense core surrounded by a bright rim were observed in the lower SC layers of all lesional samples (Fig 5B: arrowheads). Similar granules were not noted in control samples (Fig 5A and 5C).

## Discussion

Epidermal differentiation is a tightly regulated process involving multiple pathways and proteins, which are temporally and spatially expressed [11].

In this study, we show effects of derailment of this process in HNPK-affected LR carrying a *SUV39H2* genetic variant associated with an inactive SUV39H2 enzyme [5, 6]. The SUV39H2 enzyme drives primarily trimethylation of H3K9, often associated with repression of transcription of target genes [21]. Moreover, methylation of Lys on non-histone proteins is recognized as an important factor regulating important signal transduction pathways such as MAPK, WNT, BMP, Hippo and JAK–STAT [8]. These major pathways are involved in epidermal differentiation and may change terminal differentiation including altered expression of involucrin and/or loricrin. For example, activation of Yap1, a transcriptional activator of the Hippo/ YAP signaling pathway induces an increase in proliferation of basal cells in concert with an altered terminal differentiation with marked reduction in differentiation markers such as K10 and loricrin [22]. Inhibition of p38/ MAPK activity modifies terminal differentiation with decreased involucrin levels [23]. Wnt ligands such as Wnt5a promote differentiation under influence of high intracellular calcium that is characterized by increased loricrin production and decreased proliferation [24]. Dermal BMP-FGF signaling stimulated by epidermal Wnts is important for formation of the spinous layer [25]. Signal transducers and activators of transcription STAT3 and STAT6 are important regulators of keratinocyte differentiation as activation of STAT3 and STAT 6 through binding by IL-4/IL-13 decreases expression of loricrin, involucrin and filaggrin [26, 27].

In mice, *Suv39h1* and *Suv39h2* encode Suv39h methyltransferases with overlapping expression profiles during embryogenesis [28]. Whereas *Suv39h1* transcripts were present in different tissues of adult mice, *Suv39h2* transcripts were initially only detected in testis [29]. Interestingly, the phenotype of *Suv39h2* knockout mice is indistinguishable from wildtype mice [28]. Whereas the roles of the histone methyltransferases SUV39H1 and SUV39H2 have been investigated in the context of cancer in humans [30, 31] and mice [32], the role of SUV39H2 in epidermal differentiation in mice, humans and dogs is virtually unknown.

The most striking finding in this study is the complete lack of detectable loricrin by IF in HNPK-affected nasal plana. Under normal conditions, loricrin is found during the late stages of epidermal differentiation, constitutes the major component of the epidermal CE (approximately 70–85% of total protein mass) and has a key function in maintaining the structure of the cornified layer as a transglutaminase substrate [10]. Therefore, a lack of loricrin is expected to severely disrupt the terminal differentiation process and formation of the CE. This finding is corroborated by the significantly decreased loricrin gene expression in the RNA-seq analysis compared to control nasal plana.

Phenotypic consequences of loricrin deficiency have been examined in mice. Homozygous loricrin knockout mice are born with visible skin lesions such as erythroderma and shiny, translucent skin [33]. However, this phenotype normalizes 4–5 days after birth and is not associated with any histopathological abnormalities due to compensatory mechanisms [33, 34]. The existence of a similar back-up system in the dog has not yet been investigated.

Interestingly, loricrin was present in skin and paw pads of HNPK-affected dogs and its pattern was comparable to control dogs. As there are no structural differences in the epidermis between dog breeds [12, 16], we used tissue of a beagle dog as we did not have sufficient samples of LR. This finding is in line with the clinical and histological phenotype of HNPK-affected dogs where changes are restricted to the nasal planum, but no abnormalities of the haired skin or paw pads are seen.

Involucrin is an important early component of the CE, a transglutaminase substrate and marker of terminal differentiation [10]. The onset of involucrin expression in HNPK-affected dogs occurred in higher epidermal layers than in controls, pointing towards a delay in terminal differentiation in HNPK. Involucrin was also qualitatively mildly disordered in HNPK-affected nasal planum compared to non-affected nasal planum. Involucrin gene expression was significantly but less marked down-regulated, compared to loricrin. Our findings of altered involucrin expression restricted to the nasal planum are underlined by the fact that involucrin is present in paw pad and haired skin of HNPK-affected and normal dogs (S1 Fig) [12, 16].

Concerning the keratins, we could not observe significant differences in the K14, K1 and K10 epidermal distribution patterns as visualized by IF, although we detected a significant upregulation of transcripts of the major keratin genes *KRT1*, *KRT14*, and especially *KRT10* in HNPK-affected nasal planum.

RNA-seq is a method known for the risk of under- or overestimation of gene expression, especially for reads that cannot be uniquely mapped to a specific gene [35]. Of note is that the keratin protein family is well-known for high sequence homology and frequent posttranslational modifications [36]. Therefore, we used a currently curated catalog of improved keratin gene annotations [18] in order to prevent over-estimation of expression in the current study due to high sequence homology or posttranslational alterations.

This increase in keratin gene transcripts may have indeed led to an increased protein production, especially in the case of K10. Alterations in keratins are known to influence the keratinocyte cytoskeleton and through altered stiffness of the cells could have induced mechanical changes that contributed to the changes observed in H&E (pronounced irregular rete pegs) and TEM (intercellular distension and ruptures of adhesions) [37]. Dense core granules in corneocytes were typically found in lesional samples and were comparable to lipid droplets which are thought to be remnants of lamellar bodies. Comparable droplets have been described in TEM studies of parakeratosis of psoriatic epidermis [38] and Netherton syndrome [39]. These diseases are characterized by disturbed terminal epidermal differentiation associated with parakeratosis and altered lamellar body secretion [38, 39] and impaired desquamation [40].

Apparently, the loss of SUV39H2 enzyme did not alter the proliferation of keratinocytes (i.e. no significant difference in Ki-67 staining) and did not influence the formation of desmoglein 1, a transmembrane protein and constituent of desmosomes in suprabasal epidermal layers [5]. The latter observation ruled out impaired desmosome formation as an explanation for the serum lakes observed in the superficial layers.

There are some limitations to this study. Firstly, a relatively small number of dogs was included but IF microscopy results were uniform within each group on the same and on two different days, suggesting that the observations made are reproducible. Secondly, the presence of immune-mediated inflammatory cell infiltrates in the skin can influence the expression of epidermal differentiation and cornification proteins. For instance, as described in atopic dermatitis, T helper type 2-produced cytokines (especially IL-4 and IL-13) affect keratin, desmoglein, loricrin and involucrin levels [28, 41]. Inflammatory cell infiltration of the nasal planum is a feature of HNPK [1, 2] but rarely present in the nasal planum of healthy dogs [14]. In our study, cutaneous inflammation was evaluated in H&E-stained microscopic slides and considered mild to moderate in all HNPK-cases but was not assessed further.

## Conclusions

Our data suggest an altered epidermal differentiation through influence of the inactive SUV39H2 enzyme on the balanced and intricate network of signaling pathways involved in epidermal differentiation. Our findings and current knowledge on *SUV39H2* suggest a thus far unknown influence of the SUV39H2 enzyme on several genes or major pathways involved in epidermal differentiation. Further investigations are ongoing to explain why only the nasal planum is affected and will provide insight into the function of *SUV39H2* and influenced pathways involved in epidermal differentiation.

## Supporting information

S1 Text Immunofluorescence staining. (DOCX)

S1 Table List of used primary and secondary antibodies. (PDF)

S2 Table Descriptive statistics of sequence alignment. (PDF)

S1 Fig Involucrin and keratin 1 staining of paw pad and abdominal skin of non- and HNPK-affected dogs. Involucrin and K1 stainings were performed in parallel on sections of two non-affected and two HNPK-affected dogs and results were reproducible (N≥2). White hatched lines indicate the dermal-epidermal junction. Scale bars are 100μm for low magnification and 25μm for high magnification (insets).
(TIF)

## References

[1] Page N, Paradis M, Lapointe JM, Dunstan RW. Hereditary nasal parakeratosis in Labrador Retrievers Vet Dermatol. 2003; 14:103–10. 10.1046/j.1365-3164.2003.00319.x 12662268

[2] Peters J, Scott DW, Erb HN, Miller WH. Hereditary nasal parakeratosis in Labrador retrievers: 11 new cases and a retrospective study on the presence of accumulations of serum ('serum lakes') in the epidermis of parakeratotic dermatoses and inflamed nasal plana of dogs. Vet Dermatol. 2003; 14:197–203. 10.1046/j.1365-3164.2003.00341.x 12895224

[3] Bauer A, Nimmo J, Newman R, Brunner M, Welle MM, Jagannathan V, et al A splice site variant in the SUV39H2 gene in Greyhounds with nasal parakeratosis. Anim Genet. 2018; 49:137–40. 10.1111/age.12643 29423952

[4] Miller WH, Griffin CE, Campbell KE. Muller and Kirk's Small Animal Dermatology. 7th ed: Mosbey; 2013.

[5] Jagannathan V, Bannoehr J, Plattet P, Hauswirth R, Drögemuller C, Drögemuller M, et al A mutation in the SUV39H2 gene in Labrador Retrievers with hereditary nasal parakeratosis (HNPK) provides insights into the epigenetics of keratinocyte differentiation. PLoS Genet. 2013; 9:e1003848 10.1371/journal.pgen.1003848 24098150 PMC3789836

[6] Schuhmacher MK, Kudithipudi S, Kusevic D, Weirich S, Jeltsch A. Activity and specificity of the human SUV39H2 protein lysine methyltransferase. Biochim Biophys Acta. 2015; 1849:55–63. 10.1016/j.bbagrm.2014.11.005 25459750

[7] Jenuwein T, Allis CD. Translating the histone code. Science. 2001; 293(5532):1074–80. 10.1126/science.1063127 11498575

[8] Biggar KK, Li SS. Non-histone protein methylation as a regulator of cellular signalling and function. Nat Rev Mol Cell Biol. 2015; 16:5–17. 10.1038/nrm3915 25491103

[9] Helin K, Dhanak D. Chromatin proteins and modifications as drug targets. Nature. 2013; 502:480–8. 10.1038/nature12751 24153301

[10] Candi E, Schmidt R, Melino G. The cornified envelope: a model of cell death in the skin. Nat Rev Mol Cell Biol. 2005; 6):328–40. 10.1038/nrm1619 15803139

[11] Suter MM, Schulze K, Bergman W, Welle M, Roosje P, Muller EJ. The keratinocyte in epidermal renewal and defence. Vet Dermatol. 2009; 20:515–32. 10.1111/j.1365-3164.2009.00819.x 20178490

[12] Chervet L, Galichet A, McLean WH, Chen H, Suter MM, Roosje PJ, et al Missing C-terminal filaggrin expression, NFkappaB activation and hyperproliferation identify the dog as a putative model to study epidermal dysfunction in atopic dermatitis. Exp Dermatol. 2010; 19:e343–6. 10.1111/j.1600-0625.2010.01109.x 20626465

[13] Nishifuji K, Yoon JS. The stratum corneum: the rampart of the mammalian body. Vet Dermatol. 2013; 24:60–72 e15-6. 10.1111/j.1365-3164.2012.01090.x 23331681

[14] Hutt JH, Dunn KA, Scase TJ, Shipstone MA. A preliminary survey of the histopathological features of skin from the planum nasale and adjacent skin of dogs unaffected by dermatological or respiratory disease. Vet Dermatol. 2015; 26:359–62,e78–9. 10.1111/vde.12238 26189492

[15] Kobayashi T, Enomoto K, Wang YH, Yoon JS, Okamura R, Ide K, et al Epidermal structure created by canine hair follicle keratinocytes enriched with bulge cells in a three-dimensional skin equivalent model in vitro: implications for regenerative therapy of canine epidermis. Vet Dermatol. 2013; 24:77–83 e19–20. 10.1111/j.1365-3164.2012.01097.x 23331683

[16] Theerawatanasirikul S, Suriyaphol G, Thanawongnuwech R, Sailasuta A. Histologic morphology and involucrin, filaggrin, and keratin expression in normal canine skin from dogs of different breeds and coat types. J Vet Med Sci. 2012; 13:163–70. 10.4142/jvs.2012.13.2.163 22705738 PMC3386341

[17] Kim D, Pertea G, Trapnell C, Pimentel H, Kelley R, Salzberg SL. TopHat2: accurate alignment of transcriptomes in the presence of insertions, deletions and gene fusions. Genome Biol. 2013; 14:R36 10.1186/gb-2013-14-4-r36 23618408 PMC4053844

[18] Balmer P, Bauer A, Pujar S, McGarvey KM, Welle M, Galichet A, et al A curated catalog of canine and equine keratin genes. PLoS One. 2017; 12:e0180359 10.1371/journal.pone.0180359 28846680 PMC5573215

[19] Wang C, Marshall A, Zhang D, Wilson ZA. ANAP: an integrated knowledge base for Arabidopsis protein interaction network analysis. Plant Physiol. 2012; 158:1523–33. 10.1104/pp.111.192203 22345505 PMC3320167

[20] Anders S, Huber W. Differential expression analysis for sequence count data. Genome Biol. 2010; 11:R106 10.1186/gb-2010-11-10-r106 20979621 PMC3218662

[21] Rice JC, Briggs SD, Ueberheide B, Barber CM, Shabanowitz J, Hunt DF, et al Histone methyltransferases direct different degrees of methylation to define distinct chromatin domains. Mol Cell. 2003; 12:1591–8. 10.1016/s1097-2765(03)00479-9 14690610

[22] Schlegelmilch K, Mohseni M, Kirak O, Pruszak J, Rodriguez JR, Zhou D, et al Yap1 acts downstream of alpha-catenin to control epidermal proliferation. Cell. 2011; 144:782–95. 10.1016/j.cell.2011.02.031 21376238 PMC3237196

[23] Connelly JT, Mishra A, Gautrot JE, Watt FM. Shape-induced terminal differentiation of human epidermal stem cells requires p38 and is regulated by histone acetylation. PLoS One. 2011; 6:e27259 10.1371/journal.pone.0027259 22073300 PMC3206954

[24] Popp T, Steinritz D, Breit A, Deppe J, Egea V, Schmidt A, et al Wnt5a/beta-catenin signaling drives calcium-induced differentiation of human primary keratinocytes. J Invest Dermatol. 2014; 134:2183–91. 10.1038/jid.2014.149 24658506

[25] Zhu XJ, Liu Y, Dai ZM, Zhang X, Yang X, Li Y, et al BMP-FGF signaling axis mediates Wnt-induced epidermal stratification in developing mammalian skin. PLoS Genet. 2014; 10:e1004687 10.1371/journal.pgen.1004687 25329657 PMC4199507

[26] Amano W, Nakajima S, Kunugi H, Numata Y, Kitoh A, Egawa G, et al The Janus kinase inhibitor JTE-052 improves skin barrier function through suppressing signal transducer and activator of transcription 3 signaling. J Allergy Clin Immunol. 2015; 136:667–77 e7. 10.1016/j.jaci.2015.03.051 26115905

[27] Kim BE, Leung DY, Boguniewicz M, Howell MD. Loricrin and involucrin expression is down-regulated by Th2 cytokines through STAT-6. Clin Immunol. 2008; 126:332–7. 10.1016/j.clim.2007.11.006 18166499 PMC2275206

[28] Peters AH, O’Carroll D, Scherthan H, Mechtler K, Sauer S, Schofer C, et al Loss of the Suv39h histone methyltransferases impairs mammalian heterochromatin and genome stability. Cell. 2001; 107:323–37. 10.1016/s0092-8674(01)00542-6 11701123

[29] O'Carroll D, Scherthan H, Peters AH, Opravil S, Haynes AR, Laible G, et al Isolation and characterization of Suv39h2, a second histone H3 methyltransferase gene that displays testis-specific expression. Mol Cell Biol. 2000; 20:9423–33. 10.1128/mcb.20.24.9423-9433.2000 11094092 PMC102198

[30] Carvalho Alves-Silva J, do Amaral Rabello D, Oliveira Bravo M, Lucena-Araujo A, Madureira de Oliveira D, Morato de Oliveira F, et al Aberrant levels of SUV39H1 and SUV39H2 methyltransferase are associated with genomic instability in chronic lymphocytic leukemia. Environ Mol Mutagen. 2017; 58:654–61. 10.1002/em.22128 28833505

[31] Mutonga M, Tamura K, Malnassy G, Fulton N, de Albuquerque A, Hamamoto R, et al Targeting Suppressor of Variegation 3–9 Homologue 2 (SUV39H2) in Acute Lymphoblastic Leukemia (ALL). Transl Oncol. 2015; 8:368–75. 10.1016/j.tranon.2015.07.003 26500027 PMC4631083

[32] Petti E, Jordi F, Buemi V, Dinami R, Benetti R, Blasco MA, et al Altered telomere homeostasis and resistance to skin carcinogenesis in Suv39h1 transgenic mice. Cell Cycle. 2015;14(9):1438–46. 10.1080/15384101.2015.1021517 25789788 PMC4614257

[33] Koch PJ, de Viragh PA, Scharer E, Bundman D, Longley MA, Bickenbach J, et al Lessons from loricrin-deficient mice: compensatory mechanisms maintaining skin barrier function in the absence of a major cornified envelope protein. J Cell Biol. 2000; 151:389–400. 10.1083/jcb.151.2.389 11038185 PMC2192642

[34] Ishitsuka Y, Huebner AJ, Rice RH, Koch PJ, Speransky VV, Steven AC, et al Lce1 family members are Nrf2-target genes that are induced to compensate for the loss of loricrin. J Invest Dermatol. 2016; 136:1656–63. 10.1016/j.jid.2016.04.022 27167730 PMC5068579

[35] Robert C, Watson M. Errors in RNA-Seq quantification affect genes of relevance to human disease. Genome Biol. 2015; 16:177 10.1186/s13059-015-0734-x 26335491 PMC4558956

[36] Plowman JE. The proteomics of keratin proteins. J Chromatogr B Analyt Technol Biomed Life Sci. 2007; 849:181–9. 10.1016/j.jchromb.2006.07.055 16931191

[37] Ramms L, Fabris G, Windoffer R, Schwarz N, Springer R, Zhou C, et al Keratins as the main component for the mechanical integrity of keratinocytes. Proc Natl Acad Sci U S A. 2013; 110:18513–8. 10.1073/pnas.1313491110 24167246 PMC3831947

[38] Brody I. The ultrastructure of the horny layer in normal and psoriatic epidermis as revealed by electron microscopy. J Invest Dermatol. 1962; 39:519–28. 10.1038/jid.1962.151 14015700

[39] Fartasch M, Williams ML, Elias PM. Altered lamellar body secretion and stratum corneum membrane structure in Netherton syndrome: differentiation from other infantile erythrodermas and pathogenic implications. Arch Dermatol. 1999; 135:823–32. 10.1001/archderm.135.7.823 10411158

[40] Chan A, Godoy-Gijon E, Nuno-Gonzalez A, Crumrine D, Hupe M, Choi EH, et al Cellular basis of secondary infections and impaired desquamation in certain inherited ichthyoses. JAMA Dermatol. 2015; 151:285–92. 10.1001/jamadermatol.2014.3369 25565224 PMC4498571

[41] Omori-Miyake M, Yamashita M, Tsunemi Y, Kawashima M, Yagi J. In vitro assessment of IL-4- or IL-13-mediated changes in the structural components of keratinocytes in mice and humans. J Invest Dermatol. 2014; 134:1342–50. 10.1038/jid.2013.503 24280725

