# Supplementary material for: Abnormal keratinocyte differentiation in the nasal planum of Labrador Retrievers with hereditary nasal parakeratosis (HNPK)
Source: PLoS One. 2020 Mar 2;15(3):e0225901. doi: 10.1371/journal.pone.0225901 (PMC7051081; doi:10.1371/journal.pone.0225901)
Supplement: S1 Text — (DOCX) [file pone.0225901.s001.docx]

**Immunofluorescence staining**

Briefly, tissue was deparaffinized, followed by heat-mediated antigen retrieval at pH 6 in a microwave (3 x 5 min at 80 W). Slides were blocked in a moisture chamber for 90 min at room temperature with blocking buffer containing 4 % bovine serum albumin (BSA) and 0.5 % normal goat serum (NGS) in phosphate-buffered saline (PBS). The slides were incubated with the appropriate primary antibody (involucrin, Sy5, ThermoFisher Scientific, Basel, Switzerland; keratin 14, BioGenex, Fremont, CA, USA; keratin 1, Covance PRB-165P; keratin 10, Covance PRB-159P; loricrin, Covance PRB-145P; all BioLegend, San Diego, CA, USA) overnight at 4 °C using a moist tissue chamber, followed by incubation with the suitable fluorescent secondary antibody (goat anti-rabbit IgG H&L secondary antibody, Alexa Fluor® 594 conjugate; goat anti-mouse IgG H&L secondary antibody, Alexa Fluor® 488 conjugate; ThermoFisher Scientific, Basel, Switzerland) for 120 min at room temperature in the dark. The most suitable antibody dilutions were established in preliminary studies. All antibodies had been validated for their use in dogs [[1, 2](#_ENREF_10)] and the most suitable antibody dilutions were reevaluated in preliminary studies. Nuclei were counterstained with Hoechst 33258 (Sigma-Aldrich, St. Louis, MO, USA).

References

1. Kolly C, Suter MM, Muller EJ. Proliferation, cell cycle exit, and onset of terminal differentiation in cultured keratinocytes: pre-programmed pathways in control of C-Myc and Notch1 prevail over extracellular calcium signals. J Invest Dermatol. 2005;124(5):1014-25. doi: 10.1111/j.0022-202X.2005.23655.x
2. Chervet L, Galichet A, McLean WH, Chen H, Suter MM, Roosje PJ, et al. Missing C- terminal filaggrin expression, NFkappaB activation and hyperproliferation identify the dog as a putative model to study epidermal dysfunction in atopic dermatitis. Exp Dermatol. 2010; 19:e343-6. doi: 10.1111/j.1600-0625.2010.01109.x
