# Supplementary material for: Abnormal keratinocyte differentiation in the nasal planum of Labrador Retrievers with hereditary nasal parakeratosis (HNPK)
Source: PLoS One. 2020 Mar 2;15(3):e0225901. doi: 10.1371/journal.pone.0225901 (PMC7051081; doi:10.1371/journal.pone.0225901)
Supplement: S1 Table — (PDF) [file pone.0225901.s002.pdf]

S1.Table. List of used primary and secondary antibodies.

Antigen/ Clone	Reference	Antibody ID	Dilution	Host	Supplier
Loricrin	PRB-145P / Poly19051	AB_2616895	1:500	Rabbit	Covance, Biolegend[i]
Involucrin/ SY5	MA5-11803	AB 10982738	1:200	Mouse	ThermoFisher Scientific[ii]
Keratin 1	PRB-165P/ Poly19056	AB_291583	1:50	Rabbit	Covance, Biolegend[i]
Keratin 10	PRB-159P / Poly19054	AB_291580	1:500	Rabbit	Covance, Biolegend[i]
Keratin 14/ (EP61)	PRB-155P/ Poly19053	AB_292096	1:50	Rabbit	BioGenex[iii]
Secondary antibodies	Reference		Dilution	Host	Supplier
Alexa Fluor® 594 goat anti-rabbit IgG (H+L)	A-11012		1:200	Goat	ThermoFisher Scientific[ii]
Alexa Fluor® 488 goat anti-mouse IgG (H+L)	A28175		1:200	Goat	ThermoFisher Scientific[ii]

[i] BioLegend, San Diego, CA, USA

[ii] ThermoFisher Scientific, Basel, Switzerland

[iii] Biogenex, Fremont, CA, USA

Antibody ID: https://antibodyregistry.org
