# Supplementary material for: Abnormal keratinocyte differentiation in the nasal planum of Labrador Retrievers with hereditary nasal parakeratosis (HNPK)
Source: PLoS One. 2020 Mar 2;15(3):e0225901. doi: 10.1371/journal.pone.0225901 (PMC7051081; doi:10.1371/journal.pone.0225901)
Supplement: S2 Table — (PDF) [file pone.0225901.s003.pdf]

S2. Table. Descriptive statistics of sequence alignment.

	Non-affected nasal planum			HNPK-affected nasal planum		
**Sequence reads**	Dog N°3	Dog N°2	Dog N°5	Dog N°1	Dog N°4	Dog N°6
**Total reads-pairs**	58842156	68181155	69193538	69547332	55025363	58890873
**Mapped reads**	97171579 (82.6%)	53541758 (78.5%)	55526060 (80%)	114070677 (80.2%)	90276621 (80.2%)	91771621 (77.9%)
**Uniquely mapped reads**	91.7%	92.8%	90.2%	89.5%	90.7%	91.5%
**Aligned pairs**	78.8%	74.8%	76.3%	77.7%	77.8%	74.0%
