# Supplementary figures and images for: Abnormal keratinocyte differentiation in the nasal planum of Labrador Retrievers with hereditary nasal parakeratosis (HNPK)
Source: PLoS One. 2020 Mar 2;15(3):e0225901. doi: 10.1371/journal.pone.0225901 (PMC7051081; doi:10.1371/journal.pone.0225901)

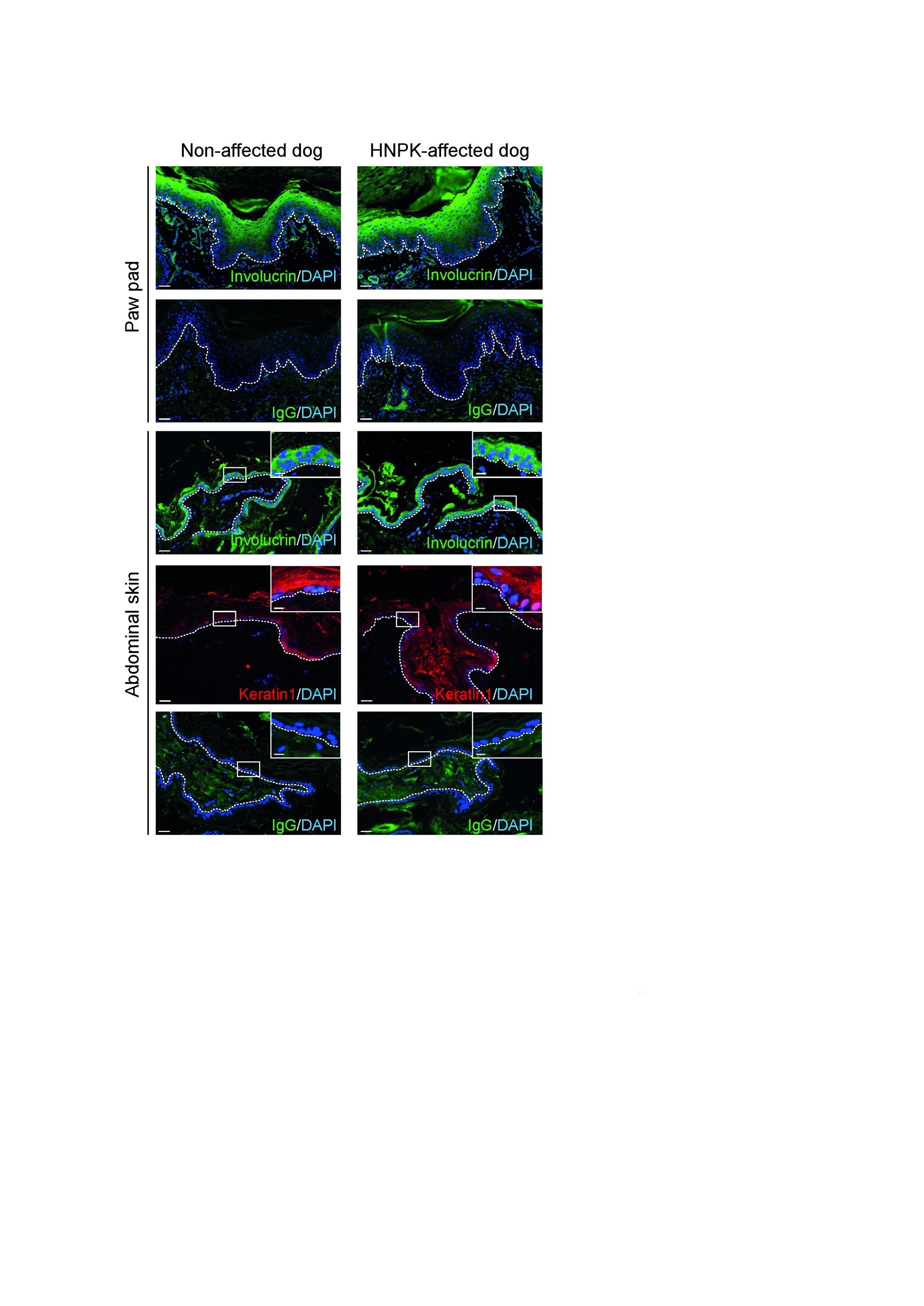

Supplement: S1 Fig — Involucrin and K1 stainings were performed in parallel on sections of two non-affected and two HNPK-affected dogs and results were reproducible (N≥2). White hatched lines indicate the dermal-epidermal junction. Scale bars are 100μm for low magnification and 25μm for high magnification (insets). (TIF) [file pone.0225901.s004.tif]
